# Editorial: Resilience in chronic disease, volume II

**DOI:** 10.3389/fpsyt.2023.1209709

**Published:** 2023-05-25

**Authors:** Zeng Jie Ye

**Affiliations:** School of Nursing, Guangzhou University of Chinese Medicine, Guangzhou, China

**Keywords:** resilience, chronic disease, vulnerability, cognition, intervention, psychosomatic

Resilience, defined as one's ability to “bounce back” from adversity, is an important attribute of patients challenged with chronic disease (1, 2). The call for submissions on Resilience in Chronic Disease: Volume I received a great response with a total of over 7,100 participants with chronic pain (3), cancer (4), irritable bowel disease (5, 6), cardiovascular disease (7), neurocognitive disorders (Wang et al.), renal transplant (8), rheumatoid arthritis (9), as well as caregivers for children with chronic illness (7), patients with liver cancer (10), and maintenance hemodialysis (7). However, most manuscript are performed with a cross-sectional design and the distinct resilience trajectories throughout the course of chronic disease are not well-explored in the first collection (11).

In *Resilience in chronic disease, volume II*, five articles and two systematic reviews with a total of over 2,000 patients and their caregivers are included. Different populations are added to this collection, including patients with stroke (Zhang et al.), breast cancer (Liang et al.), cervical spondylosis (Chu et al.), and disabilities (Abulaiti et al.) as well as pregnant women (Mei et al.). Both cross-sectional and longitudinal designs are utilized in these manuscripts and quantitative and qualitative data are also well-analyzed. In addition, one review conceptualizes the concept of suicide resilience (Wang et al.) and another review summarizes the effect of life review on psychospiritual outcomes among older adults with life-threatening illnesses (Liu et al.). These manuscripts provide insights to raise awareness as well as reduce the detrimental impact of different chronic disease on patients and their caregivers. However, several limitations still remain and should be further addressed in future collections. First, new resilience theory or instrument are still missing and the debate about resilience construction cannot be further advanced (12–14). Second, as for studies designed for associations between resilience and other psychosocial outcomes in different populations, measurement errors should be taken into consideration especially in the moderated mediation analysis. Third, several advanced methods including latent profile analysis and latent growth mixed model can be performed to explore heterogeneity in cross-sectional and longitudinal studies respectively, which can provide the robustness of the conclusion (15). Fourth, most resilience instruments and other patient reported outcomes (PROs) are based on Classical Test Theory (CTT) and its hypothesis of linear association between score and ability is not feasible in real world. Thus, several advanced psychometric methods (i.e., multidimensional item response theory, cognitive diagnosis model, etc.) can be utilized to get a more accurate estimation of ability compared to a total score (16–18). Fifth, intervention research designed for enhancing resilience and resilience-related PROs in patients with chronic disease and their caregivers are still scarce in this collection and the efficacy, sustainability and implementation challenges of resilience programs in patient with different chronic diseases should be further explained (19–22). At last, multi-modal and cross-modal methods including Functional Magnetic Resonance Imaging, Positron Emission Computed Tomography, Intracranial Electroencephalography, Magnetoencephalography, etc., can be performed to explore the associations between resilience and brain functions or structure (23, 24).

## Author contributions

The author confirms being the sole contributor of this work and has approved it for publication.
